# The Cardiac Power Index during Abdominal Open Aortic Surgery: Intraoperative Insights into the Cardiac Performance—A Retrospective Observational Analysis

**DOI:** 10.3390/jpm12101705

**Published:** 2022-10-12

**Authors:** Fulvio Nisi, Enrico Giustiniano, Massimo Meco, Luca Pugliese, Lorenzo Calabrò, Sofia Spano, Umberto Ripani, Maurizio Cecconi

**Affiliations:** 1 Department of Anesthesia, Intensive Care Unit and Pain Therapy, IRCCS Humanitas Clinical and Research Center, Via Manzoni 56, 20089 Milan, Italy; 2 Department of Anesthesia and Intensive Care, Humanitas Gavazzeni Clinics, Via Mauro Gavazzeni 21, 24125 Bergamo, Italy; 3 Department of Biomedical Sciences, Humanitas University, Via Rita Levi Montalcini 4, 20090 Milan, Italy; 4 Division of Clinic Anaesthesia, Department of Emergency Hospital Riuniti, Conca Street 71, 60126 Ancona, Italy

**Keywords:** cardiac output, hemodynamic monitoring, ventriculo-arterial coupling, aortic open repair, vascular surgery

## Abstract

Background: The Cardiac Power Index (CPI) measures the rate of energy output generated by the heart and correlates this with in-hospital mortality due to cardiogenic shock. In open aortic surgery, both aortic clamping and unclamping expose the heart to abrupt variations of the left ventricle afterload, preload, and contractility, with possible hemodynamic impairment. We investigated how aortic-cross clamping (Ao-XC) and unclamping (Ao-UC) procedures affect the CPI during open aortic surgery. Methods: We retrospectively analyzed our surgical database of 67 patients submitted to open surgical aortic repair at Humanitas Research Hospital, Milan. Patients were monitored by an EV1000-FloTrac System^TM^ (Edwards Lifescience, Irvine, CA, USA) beyond the standard intra-operative hemodynamic monitoring. The primary outcome was the variation of basal CPI after aortic clamping and unclamping. Secondary outcomes were variations of the cardiac index (CI), mean arterial pressure (MAP), heart rate, and lactate during aortic clamping and after unclamping. The CPI was computed as: (CI × MAP)/451. Results: The CPI changed significantly after aortic unclamping. CPI: basal = 0.39 ± 0.1 W/m^2^, after Ao-XC = 0.39 ± 0.1 W/m^2^, and after Ao-UC = 0.44 ± 0.2 W/m^2^, *p* < 0.05. The CI changed during both cross-clamping and unclamping (*p* < 0.0001), whilst the MAP and heart rate did not during any phase of the surgery. Five subjects (8.3%) needed inotropic support after cross-clamping. Their basal CPI was lower than the general population: 0.31 ± 0.11 W/m^2^ vs. 0.39 ± 0.1 W/m^2^. Conclusions: The CPI describes the adaptation of the cardiac function to the changes in preload, contractility, and afterload occurring during aortic cross-clamping and unclamping. It may be used to explore the cardiac performance in real-time and predict cardiac impairment in the intraoperative period in a minimally invasive way, similar to ventriculo-arterial coupling parameters.

## 1. Introduction

Open aortic surgery is a major operation that exposes the patient both to the risks associated with major surgery and to hemodynamic impairment related to the phases of aortic cross-clamping (Ao-XC) and unclamping (Ao-UC). Both aortic clamping and unclamping, particularly if supra-renal or supra-celiac, may be associated with renal, hepatic, and intestinal ischemia [1,2]. These may occur due to the interrupted splanchnic blood flow as well as to both the reperfusion injury (after unclamping) and the low-flow state causing a mismatch between the tissue need and the delivery of oxygen (DO_2_). Moreover, the low-flow state itself may occur due to the inability of the heart to cope with the increased afterload after the aorta cross-clamping or to the reduced effective blood volume after the restoration of the blood flow once the clamp is removed [3]. In addition, blood volume redistribution during clamping may exacerbate cardiac impairment or favor cardiac function during these phases. The ability of the heart to withstand increased work and maintain stable perfusion pressure is fundamental to maintaining balance in the cardiovascular system during surgery. Research on the topic of blood pressure control and intraoperative hypotension prevention during surgery has produced relevant evidence in recent years. Indeed, intra-operative blood pressure management has been described as an issue of utmost importance since hypotension, even when occurring briefly, has been proven to be related to postoperative complications [4,5].

The cardiac power output (CPO) is a mathematical index combining two parameters of heart physiology, flow and pressure, i.e., the cardiac output (CO) and mean arterial blood pressure (MAP). It has been described as the strongest marker that independently correlates with the in-hospital mortality due to cardiogenic shock. Moreover, it has also been found to be inversely correlated with the age of the patient [6,7]. Indexation of the CPO by the body surface area (BSA) leads to the Cardiac Power Index (CPI). As a reference value for the CPI, Vincent (2009) reported a normal value as being in the range between 0.5 and 0.7 W/m^2^, even though they suggested considering its trend rather than any isolated value or outlier in clinical decision-making [8].

Our primary aim was to assess how aortic-cross clamping and unclamping procedures affect CPI during open aortic surgery. Our secondary aim was to assess how aortic cross-clamping/unclamping influences other hemodynamic values such as the MAP, cardiac index (CI), and heart rate (HR).

## 2. Materials and Methods

We retrospectively analyzed our surgical database of patients undergoing open abdominal aortic surgical repair during the years 2020–2021.

Patients’ informed consent was obtained and the study received the approval of our institutional ethical committee (EC appr. 10/21).

For this observational study, we followed the STROBE (Strengthening the Reporting of Observational Studies in Epidemiology) statement recommendations [9].

The primary outcome measure of the study was the variation of basal CPI after aortic clamping and unclamping. Secondary outcome measures were the variations of CI, MAP, HR, and lactate during aortic clamping and after unclamping.

### 2.1. Intraoperative Monitoring and Management

According to our institutional protocol, all patients received general anesthesia (fentanyl, midazolam, and propofol for induction; sevoflurane and remifentanil for maintenance; rocuronium for myorelaxation). All patients were routinely monitored with the semi-invasive EV1000-FloTrac System^TM^ (Edwards Lifescience, Irwin, CA, USA) alongside standard intra-operative monitoring (electrocardiogram D1 and V5, invasive blood pressure, end-tidal carbon dioxide and peripheral oxygen saturation). The radial artery was cannulated in 100% of patients for invasive monitoring. The cardiac power output was computed from both the cardiac output (CO) and mean arterial blood pressure according to the following formula:

CPO = (CO × MAP)/451    (unit of measure Watts, W)(1)

Aiming to obtain data tailored to each patient, the cardiac index replaced the CO in the formula, thus obtaining the Cardiac Power Index [6]:

CPI = (CI × MAP)/451  [unit of measure Watts/m^2^, W/m^2^] (2)

Regarding respiratory management, we set the mechanical ventilation aiming for a tidal volume of 4–5 mL/kg of the predicted body weight (PBW), PEEP 5 cmH_2_O, I:E = 1:2, and a respiratory rate set to achieve 30–40 mmHg etCO_2_ (end-tidal CO_2_). According to our previous report [10], if the patient tolerated a positive end-expiratory pressure (PEEP) > 5 cmH_2_O during mechanical ventilation (i.e., peak airway pressure < 35 mmHg), then during the clamping phase, we would increase the PEEP to 10 cmH_2_O (maintaining a tidal volume of 4–5 mL/kg). Whilst unclamping, we reset the PEEP to zero (ZEEP), held it for 1 min, and then raised the PEEP back to 5 cmH_2_O. These maneuvers aimed at limiting blood pressure variations due to clamping and unclamping by enhancing venous return to the right heart.

Hemodynamics was managed using a stroke volume variation (SVV) target (usually 10–15%) before clamping or after unclamping, whilst during the aortic clamping phase, we referred to MAP and Stroke Volume Index (SVI) values. In case of a need for further cardiac performance assessment, we used trans-esophageal echocardiography (TEE). Our hemodynamic targets were generally MAP > 65 mmHg and CI > 2 L/min/m^2^. The heart rate target was the rate the patient showed at the preoperative electrocardiogram, and if it Increased by >20–30% (in any case >95 beats/min), the patient would receive a short-acting β_1_-blocker, according to the anesthesiologist’s clinical judgment. When the patient needed a vasoconstrictive drug, our first choice was norepinephrine (NE); as an inotropic support, we used dobutamine (DB) if necessary.

With regard to fluid therapy, we administered balanced crystalloids of 4 mL/kg/h; further fluid input was guided by targeting SVI or SVV. If SVV > 15%, a fluid bolus of 4 mL/kg/5 min was administered as a fluid challenge (no more than three times). If a colloid solution was necessary (i.e., during bleeding, aiming at limiting the volume of crystalloid loading), 20% albumin solution was used.

Blood losses were replaced by blood from the red cell saver as the first choice. If the result was insufficient, we administered concentrated red blood cells and fresh frozen plasma from our Institutional Blood Bank. Our transfusion targets were Hb 8–10 g/dL for patients with acute coronary syndrome or chronic ischemic disease and Hb < 7 mg/dL for all other patients.

According to the anesthetist’s judgment, antioxidant therapy with an N-acetyl-cysteine (NAC) 15 mg/kg bolus was administered before aortic cross-clamping.

For postoperative pain control, all subjects received 0.4% ropivacaine through a supra-fascial catheter inserted by the surgeon at the end of the operation, after he had infiltrated the abdominal fascia itself with 0.375% ropivacaine + 1% lidocaine at the beginning of the operation.

According to an institutional fast-track protocol, all the patients routinely received a hypercaloric drink in the morning, at least two hours before their admission to the operating theatre. When the patient returned to the ward after the operation, usually in the evening (at least 4 h after awakening), he received oral fluids (usually a cup of tea) and assisted early mobilization (sitting on an armchair, then later standing up and having a brief walk) [11].

### 2.2. Data Collection

Hemodynamic data were collected throughout the surgical procedures. We recorded the CI and MAP and computed the CPI before aortic clamping (basal), 5 min after aortic cross-clamping, and 5 min after unclamping. Blood gas analysis (BGA) was routinely performed before Ao-XC, just before the Ao-UC, and 15 min after the Ao-UC, and serum lactate concentrations were obtained.

The hemodynamic effects of the PEEP levels were evaluated by measuring the MAP, CI, and CPI during and after aortic cross-clamping. Intraoperative diuresis, the serum lactate concentration, blood loss, blood product transfusions and diuretics needed were recorded.

The data on the postoperative outcome that we recorded were the rate of extubation at the end of surgery, need for and duration of postoperative respiratory support, rate of postoperative Intensive Care Unit (ICU) admission, rates and types of complications, and ICU and hospital length of stay. The pre-operative and 48-h postoperative serum creatinine were compared as a marker of acute kidney injury (AKI). Furthermore, we considered any adverse cardiac event, respiratory impairment requiring ICU admission, postoperative bleeding due to suture leakage, gastrointestinal dysfunction, and cerebrovascular events. Finally, we searched for a minor transient alteration of blood markers postoperatively in the absence of evidence of organ injury: serum lactate, C-reactive protein, and serum troponin.

### 2.3. Statistics

Data are reported as the mean ± standard deviation or median and interquartile range (IQR) or number and percentage, as appropriate.

We used ANOVA or Student’s *t*-test for parametric variables and the Mann–Whitney U test for non-parametric data, as appropriate.

Spearman’s correlation test was used to explore the association between the CPI and other parameters. A Spearman’s coefficient (r) between 0 and 0.25 showed no or a low correlation, 0.25 to 0.5 a decent correlation, 0.5 to 0.75 a moderate to good correlation, and 0.75 to 1 a very good to excellent correlation. Spearman’s coefficient was reported with its 95% confidence interval. If any correlation was found to be relevant, then a linear regression analysis was performed. An obtained *p*-value < 0.05 was considered statistically significant.

Analysis was performed with Prism 8.2.1 Software (GraphPad, San Diego, CA, USA).

## 3. Results

Between January 2020 and December 2021, we observed 67 patients who underwent open surgical aortic repair for aneurysm. Of those, seven subjects were excluded because the data were incomplete (Supplemental Figure S1), meaning the final sample consisted of 60 cases. Characteristics of the sample are reported in Table 1.

Intra-operative data on the cardiovascular performance are summarized in Table 2.

**Figure 1:**
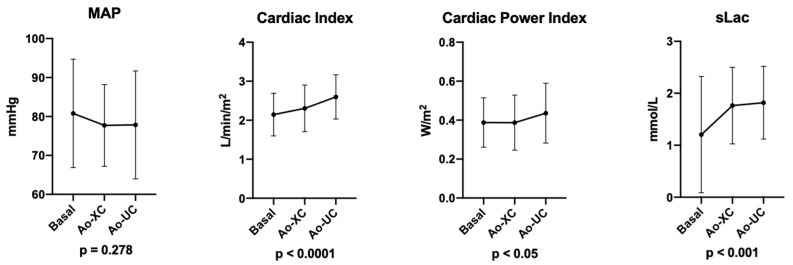
Intraoperative cardiovascular performance. MAP, cardiac indexm and Cardiac Power Index time-frame recordings were: basal (i.e., before the aortic clamping), Ao-XC (5 min after aortic cross-clamping), and Ao-UC (5 min after unclamping). Serum lactate concentrations were measured: basal (i.e., before aortic clamping), Ao-XC (5 min before the unclamping), and Ao-UC (15 min after the aortic unclamping). ANOVA with repeated measures was performed. Abbreviations: MAP, mean arterial pressure; sLac, serum lactate concentration; Ao-XC, aortic cross-clamping; Ao-UC, aortic unclamping.

The Cardiac Power Index changed significantly during the procedure, especially after aortic unclamping. In particular, the basal CPI = 0.39 ± 0.1 W/m^2^, CPI after Ao-XC = 0.39 ± 0.1 W/m^2^, and CPI after Ao-UC rose to 0.44 ± 0.2 W/m^2^ (*p* < 0.05) (Figure 1).

The cardiac index increased significantly after aortic clamping and unclamping: basal CI = 2.15 ± 0.6 L/min/m^2^, after Ao-XC CI = 2.31 ± 0.6 L/min/m^2^, and after Ao-UC CI = 2.6 ± 0.6 L/min/m^2^, *p* < 0.0001.

The heart rate and MAP failed to change significantly after aortic clamping and unclamping. After Ao-UC, 36 patients (60.0%) needed NE administration to maintain their MAP ≥ 65 mmHg. NE administration was always stopped before the end of the operation since safe hemodynamics were restored in all cases. Five subjects (8.3%) needed dobutamine (DB) administration after Ao-XC. Their average CPI was 0.31 ± 0.11 W/m^2^, CI was 1.7 ± 0.4 L/min/m^2^, MAP was 78.2 ± 13.4 mmHg, and HR was 50.0 (48.0÷58.0) bpm. Trans-esophageal echocardiographic exams confirmed the significant reduction in global systolic function, whilst none of them reported regional wall motion abnormalities. After aortic unclamping, DB was progressively weaned because the hemodynamics improved.

Subanalysis in patients with and without the need for vasoactive drugs during surgery showed similar results, both in terms of the blood pressure levels and the cardiac performance.

Unsurprisingly, serum lactate concentrations (sLac) changed significantly after clamping and unclamping (*p* < 0.001) (Table 2).

Among the main cardiovascular risk factors (age, smoking, hypertension, diabetes, and history of heart failure) potentially affecting CPI, only age showed a significant correlation (Supplemental Table S1). Similar to previous reports [6,7], the basal CPI showed an inverse linear correlation with age (Supplemental Figure S2).

The CPI variation, i.e., the difference between Ao-XC and Ao-UC, did not show any significant correlation with any of the preoperative risk factors or with the intraoperative PEEP level during aortic clamping (Supplemental Table S1).

Intraoperative fluid therapy and blood management data are reported in Supplemental Table S2. Antioxidant therapy was administered in 53 cases.

Postoperative data on the outcome, hospital length-of-stay, and complications are reported in Supplemental Table S3.

In a subanalysis, we divided the sample according to the PEEP level during the clamping phase: PEEP 5 cmH_2_O, or PEEP 10 cm_2_O (Supplemental Table S4). We obtained two subgroups: PEEP 10 cmH_2_O (N = 38, subgroup A), and PEEP 5 cmH_2_O (N = 22, subgroup B). After aortic unclamping and restoration of the blood flow to the splanchnic circulation, MAP variation did not differ significantly between the two subgroups, with no difference recorded in the need for vasoactive drugs between the two subgroups. Similarly, the cardiac index did not seem significantly influenced by the PEEP level, nor was the CPI.

## 4. Discussion

The main finding of our study is that the CPI described the adaptation of the cardiac function to the changes in preload or afterload occurring during aortic cross-clamping and unclamping. According to Gelman [3], aortic clamping produces an increase in afterload and a shift of blood volume proximally to the clamp and into the splanchnic vasculature, which may increase the venous return and preload (or sometimes reduce the preload if the splanchnic venous tone is low). When unclamping, a reduction in myocardial contractility occurs secondary to mediator release from the now re-perfused splanchnic district. Moreover, an increase in venous capacitance, distal shift of blood, and central hypovolemia lead to reduced venous return, cardiac output, and hypotension.

In our population, 83.4% of the patients underwent infra-coeliac clamping. Independently of the level of aortic clamping, during the cross-clamping, there was an increase in left ventricle stroke volume, leading to increased CI, whilst the expected increase in afterload did not affect the cardiac ability to maintain a stable perfusion pressure, as suggested by a stable value of MAP. In all patients, presumably, the increase in the cardiac index was driven by an increase in contractility and preload rather than an increase in the heart rate, which did not undergo any significant change.

Compared to Gelman’s physiology thesis [3], when unclamping, our patients did not experience any significant variation in MAP or systemic hypotension, although a distal blood shift and a reduction in afterload presumably occurred. We hypothesized that several intertwined corrective actions carried out by the anesthesiologist avoided the occurrence of hypotension and ensured an increase in the preload, cardiac index, and cardiac power. In this regard, norepinephrine administration to sustain arterial tone, acidosis correction, fluid administration, and PEEP zeroing [12,13] supposedly played a pivotal role, together with dobutamine administration (in a small subgroup of patients). Hence, the cardiac power increase, in this condition, represented an increased efficiency of the system, able to generate more power when required.

Compared with the normal values described by Vincent et al. [8], in our population, the basal CPI result was lower, showing a value just below the range of normality. This is reasonable under general anesthesia, when both basal metabolism and energy expenditure are lower than at normal rest.

Under normal conditions, the heart pumps close to its maximal efficiency and maximal power, with an efficiency of about 25% [14]. According to Squara et al., any failing heart has a predetermined CPO, depending on the myocardial oxygen delivery and global heart pump efficiency [15]. Thus, cardiac power defines the balance or imbalance of the cardiovascular system, describing adaptation of the heart function to changes in flow and pressure, i.e., through the description of the mutual variations of BP and CO. Indeed, in a healthy heart, when the blood pressure (BP) increases, the CO may decrease, and cardiac power remains stable; otherwise, the increased BP can provide better coronary flow and the heart pumps more efficiently. The net result is an increase in CO and cardiac power [15]. If the heart is already at its maximal cardiac power, when blood pressure increases, CO decreases to maintain efficiency in the cardiovascular system. Up to some point, the system will maintain this balance. Eventually, depending on the extent or the duration of this condition, CO decrease will be deleterious, leading to decompensation and cardiac power reduction. Neither the single CO nor MAP values will be of help in such a condition, but CPI computing can inform the physician when this decompensation is occurring.

Noteworthy, even though most patients were ASA II or III class and almost one-third (28.3%) had chronic ischemic cardiomyopathy, the compensatory cardiovascular mechanisms reacting to cross-clamping/unclamping were intact or at a rate able to withstand sustained injury for the time necessary, if pharmacologically supported, which was a sign of an overall healthy heart, closely following the physiological Frank–Starling curve. Indeed, only a very small percentage of patients needed inotropic support with dobutamine during critical phases. This same population presented a reduced basal CPI (0.31 ± 0.11 W/m^2^) compared to our general population (0.39 ± 0.1 W/m^2^).

Intrinsic in nature, the CPI is meant to be an indicator of the efficiency of cardiac contractility and ventriculo-arterial coupling [14]. In a previous study, William et al. [16] reported that cardiac power is the strongest predictor of mortality in patients suffering from cardiogenic shock, and showed that levosimendan increases CPI better than dobutamine, without a significant change in pulmonary capillary wedge pressure in subjects with myocardial infarction. In our sample, only five patients received dobutamine, which was effective at restoring stable hemodynamics. A collateral finding of our analysis is that CPI decreases with increasing age, this being consistent with reduced cardiovascular performance in the elderly, as previously reported by William et al. [16].

Although cardiac power is a rather abstract measure, computing the basal CPI before the surgical incision and following its changes during surgery may be useful to explore cardiac performance in real-time and predict cardiac impairment in the intraoperative period. Compared to ventriculo-arterial coupling parameters (such as left ventricular end-systolic elastance/arterial elastance, Ees/Ea) [17] or trans-esophageal echocardiography, which requires the availability of intraoperative ultrasounds, measurements to derive CPI are easy to obtain by means of pulse wave analysis in a continuous, minimally invasive (or even noninvasive) manner. Pulse contour analysis is indeed feasible during almost every kind of surgery in which an arterial line is used. Nevertheless, echocardiography remains the gold standard for cardiac function assessment due to its ability to provide multiple pieces of information and allow a precise diagnosis of sudden cardiac dysfunction in order to guide treatment.

Our study had several limitations. First, it was a retrospective observational trial. Second, the lack of preload and afterload direct measurements, together with the relatively small sample, meant the study lacked the power for us to draw definite conclusions. Indeed, the direction (increase or decrease) of changes in the preload and afterload could be correlated with the CPI variations, but the extent of changes was not directly measured. This may limit the validity of our conclusions. In addition, differently from Ees/Ea, the CPI is not a load-independent parameter, this being in common with the echocardiography-derived ejection fraction. In this regard, dependency on CI, i.e., on the stroke volume, means preload dependency. Unfortunately, data on direct measurements of the preload and afterload were incomplete in our database and so we did not include them in the analysis.

Third, only five patients underwent TEE evaluation during the operation and subsequent dobutamine administration. A routine intraoperative ultrasound evaluation of the cardiac performance would have added useful insights and made our conclusions more realistic. Fourth, we monitored hemodynamics by means of a semi-invasive pulse contour analyzing device, despite it having received criticism about its employment during abdominal aortic surgery [18]. In the report in question, Maeda et al. compared CI measured by FloTrac/Vigileo™ with CI measured by 3D echocardiography. We agree that FloTrac/Vigileo™ may not be as reliable as other monitoring systems in this setting. Nevertheless, among its advantages, it is readily available and can provide useful information in a continuous manner throughout the surgery. Furthermore, we used the latest-generation FloTrac/EV1000 device, which benefits from software updates that have improved the reliability of the tool over time [19].

## 5. Conclusions

In conclusion, in open surgery of the abdominal aorta, the Cardiac Power Index seemed to reliably assess the intraoperative performance of the heart pump function. This leads us to propose that it may represent a minimally invasive intraoperative measurement of ventriculo-arterial coupling. In the future, a prospective randomized trial including direct intraoperative cardiac assessment is needed to draw definite conclusions.

## Figures and Tables

**Table 1:** Study population.

Title 1	N (%)
Sample size	60 (100)
Age (years)	71.2 ± 9.0
Male sex	55 (91.6)
BMI (kg/m^2^)	26.4 ± 3.8
ASA score	
1	1 (1.6)
2	23 (38.4)
3	36 (60.0)
Co-morbidities and risk factors	
Hypertension	54 (90.0)
Smoker	37 (61.6)
Chronic ischemic cardiomyopathy	17 (28.3)
Cardiac rhythm disorder	9 (15.0)
Diabetes mellitus	5 (8.3)
Chronic obstructive pulmonary disease	11 (18.3)
Liver disease	5 (8.3)
Obesity ^a^	9 (15.0)
Chronic renal failure ^b^	6 (10.0)
Peripheral vascular disease ^c^	1 (1.6)
Chronic cerebrovascular disease	4 (6.7)
Pre- and intra-operative data	
Pre-operative β-blocker therapy	29 (48.3)
Pre-operative serum creatinine (mg/dL)	1.01 ± 0.30
Type of aortic repair	
Infra-renal aortic tract repair	50 (83.4)
Supra/Juxta-renal aortic tract repair	10 (16.6)
Intraoperative antioxidant (N-acetyl-cysteine)	53 (88.3)

Data are reported as a number (N; percentage, %) or mean ± SD or median (IQR) as appropriate. ^a^ No obstructive sleep apnea syndrome. ^b^ No renal replacement therapy. ^c^ Any degree of peripheral vascular disease requiring previous surgical treatment. Abbreviations: BMI, body mass index; ASA, American Society of Anesthesiologists.

**Table 2:** Intraoperative cardiovascular performance.

	**Basal**	**Ao-XC ^a^**	**Ao-UC ^b^**	***p***
Heart rate (bpm)	61.5 (55.3 ÷ 70.0)	59.0 (52.5 ÷ 69.5)	61.0 (55.3 ÷ 72.8)	0.409
Mean arterial pressure (mmHg)	80.8 ± 13.9	77.7 ± 10.5	77.8 ± 13.9	0.278
Cardiac index (L/min/m^2^)	2.15 ± 0.6	2.31 ± 0.6	2.6 ± 0.6	<0.0001
Cardiac Power Index (W/m^2^)	0.39 ± 0.1	0.39 ± 0.1	0.44 ± 0.2	<0.05
	**Basal**	**Ao-XC ^c^**	**Ao-UC ^d^**	***p***
Serum lactate (mmol/L)	1.0 ± 0.3	1.8 ± 0.7	1.8 ± 0.7	<0.001

Data are reported as the mean ± SD or median (IQR) as appropriate. ANOVA with repeated measures was performed. ^a^ 5 min after Ao-XC; ^b^ 5 min after Ao-UC; ^c^ 5 min before Ao-UC; ^d^ 15 min after Ao-UC. Abbreviations: Ao-XC, aortic cross-clamping; Ao-UC, aortic unclamping.

## Supplementary Materials

The following supporting information can be downloaded at: https://www.mdpi.com/article/10.3390/jpm12101705/s1, Figure S1: Study flow chart; Figure S2: CPI and age; Table S1: Multiple correlations of CPI and CPI variation with cardiovascular risk factors; Table S2: Intra-operative fluid therapy; Table S3: Postoperative and outcome data; Table S4: Effect of PEEP level during aortic clamping on hemodynamics and outcome.
