# Supplementary material for: The Cardiac Power Index during Abdominal Open Aortic Surgery: Intraoperative Insights into the Cardiac Performance—A Retrospective Observational Analysis
Source: J Pers Med. 2022 Oct 12;12(10):1705. doi: 10.3390/jpm12101705 (PMC9605046; doi:10.3390/jpm12101705)
Supplement: Supplementary file 1 [file jpm-12-01705-s001.zip › Supplemental Table S1.pdf]

Supplemental Table S1 – Multiple correlations of CPI and CPI variation with cardiovascular risk factors

	Variable	R	95% CI	p
**CPI**				
	Age	-0.271	-0.497 to -0.011	0.036
	Smoking	0.445	0.208 to 0.632	0.480
	Hypertension	-0.146	-0.392 to 0.120	0.266
	Diabetes	-0.136	-0.384 to 0.130	0.299
	CHF	-0.224	-0.458 to 0.040	0.086
**CPI variation**†				
	Age	0.188	-0.077 to 0.428	0.151
	Smoking	-0.001	-0.270 to 0.252	0.940
	Hypertension	-0.071	-0.326 to 0.194	0.592
	Diabetes	-0.088	-0.341 to 0.177	0.502
	CHF	0.201	-0.063 to 0.439	0.123
	PEEP level§	-0.164	-0.408 to 0.102	0.211

Spearman r correlation performed.
†CPI variation was computed as the difference in CPI between aortic cross-clamping and unclamping (W/m^2)
§PEEP level is intended as the level of PEEP applied during the clamping phase.
Abbreviations.
CPI, Cardiac Power Index; CHF, history of Cardiac Heart Failure; PEEP, positive end-expiratory pressure
