# Supplementary material for: The Cardiac Power Index during Abdominal Open Aortic Surgery: Intraoperative Insights into the Cardiac Performance—A Retrospective Observational Analysis
Source: J Pers Med. 2022 Oct 12;12(10):1705. doi: 10.3390/jpm12101705 (PMC9605046; doi:10.3390/jpm12101705)
Supplement: Supplementary file 1 [file jpm-12-01705-s001.zip › Supplemental Table S2.pdf]

Supplemental Table S2– Intra-operative fluid therapy

	n (%)	ml/kg	Units/patient
Diuresis during aortic clamping		1.08±0.8	
Furosemide administration	5 (8.3)		
Blood losses		11.5±6.4	
Blood Products transfusion			
Blood from red cells saver	43 (71.7)	9.2±5.2	
Concentrated Red Blood Cells	4 (6.7)	5.4±3.1	1.8
Fresh Frozen Plasma	1 (1.6)	6.4±0.0	2
Albumin 20% solution	40 (66.7)		2

Data are reported as number, N (percentage, %) or mean ± SD or median (IQR) as appropriate.
