# Supplementary material for: The Cardiac Power Index during Abdominal Open Aortic Surgery: Intraoperative Insights into the Cardiac Performance—A Retrospective Observational Analysis
Source: J Pers Med. 2022 Oct 12;12(10):1705. doi: 10.3390/jpm12101705 (PMC9605046; doi:10.3390/jpm12101705)
Supplement: Supplementary file 1 [file jpm-12-01705-s001.zip › Supplemental Table S3.pdf]

## Supplemental Table S3 – Postoperative and Outcome data

| | N (%) | Median (IQR) | Mean±SD |
|---|---|---|---|
| Extubated at the end of surgery | 58 (96.7) | | |
| ICU admission | 4 (6.7) | | |
| Post-operative invasive mechanical ventilation | 2 (3.3) | | |
| Duration of respiratory support (hours) | | | 8±2.8 |
| ICU length-of-stay (days) [b] | | | 1±0.0 |
| Hospital length-of-stay (days) [b] | | 3 (3÷4) | |
| Complications | | | |
| Major complications: | 20 (33.3) | | |
| Ischemic cardiac injury | 1 (1.6) | | |
| Respiratory impairment | 8 (13.3) | | |
| Bleeding | 0 (0) | | |
| Gastro-intestinal injury | 2 (3.3) | | |
| Renal injury | 6 (10.0) | | |
| Cerebro-vascular events | 0 (0) | | |
| Fever or signs of infection | 2 (3.3) | | |
| Lower limb ischemia | 1 (1.6) | | |
| Other Minor complications[c] | 14 (23.3) | | |
| Deaths | 0 (0) | | |

| | Pre-operative | 48h post-operative | p |
|---|---|---|---|
| Serum Creatinine (mg/dL)[a] | 0.94 (0.8÷1.1) | 1.0 (0.8÷1.2) | 0.037 |
| Antioxidant therapy (N=53)[a] | 0.94 (0.8÷1.1) | 1.02 (0.8÷1.2) | 0.027 |
| No antioxidant therapy (N=7)[a] | 1.07 (0.8÷1.1) | 1.00 (0.7÷1.2) | 0.999 |

Measures are reported as number, N (percentage, %)) or mean ± SD or median (IQR) as appropriate.

[a] Wilcoxon rank test performed

[b] Only one patient experienced a complication not related to the surgery and stayed in Hospital >30 days.

[c] Minor transient complications: C-Reactive protein increasing >10mg/dL (n=2); in-ward Serum Lactate >4 mmol/L (n=5); Serum Troponin increasing without cardiac injury (n=1); Abdominal wall hematoma (n=1).
