# Supplementary material for: The Cardiac Power Index during Abdominal Open Aortic Surgery: Intraoperative Insights into the Cardiac Performance—A Retrospective Observational Analysis
Source: J Pers Med. 2022 Oct 12;12(10):1705. doi: 10.3390/jpm12101705 (PMC9605046; doi:10.3390/jpm12101705)
Supplement: Supplementary file 1 [file jpm-12-01705-s001.zip › Supplemental Table S4.pdf]

Supplemental Table S4 – Effect of PEEP level during Aortic clamping on hemodynamics and outcome

	PEEP 10 (N=38)	PEEP 5 (N=22)	p
BMI (kg/m^2)	26.7±3.7	26.0±4.1	0.527
MAP basal (mmHg)	82.4±14.3	78.1±13.0	0.247
MAP at Ao Clamp (mmHg)	76.4±11.4	80.2±8.1	0.192
MAP at Ao Unclamp (mmHg)	79.0±13.2	75.8±15.2	0.408
MAP difference between Ao-XC and Ao-UC (mmHg)	-2.55±17.4	4.05±14.7	0.140
Δ MAP between Ao-XC and Ao-UC[b]	0.1±0.23	-0.1±0.19	0.086
CI basal (L/min/m^2)	2.2±0.6	2.2±0.4	0.948
CI at Ao Clamp (L/min/m^2)	2.2±0.7	2.5±0.4	0.168
CI at Ao Unclamp (L/min/m^2)	2.5±0.6	2.8±0.5	0.061
Δ CI between Ao-XC and Ao-UC[c]	0.2±0.2	0.1±0.2	0.852
CPI basal (W/m^2)	0.40±0.1	0.37±0.1	0.448
CPI at Ao Clamp (W/m^2)	0.38±0.1	0.40±0.2	0.636
CPI at Ao Unclamp (W/m^2)	0.44±0.1	0.43±0.2	0.741
CPI difference between Ao-XC and Ao-UC (W/m^2)	-0.1±0.1	-0.0±0.1	0.339
Δ CPI between Ao-XC and Ao-UC[d]	-0.2±0.4	-0.1±0.3	0.129
Norepinephrine administration; N (%)[a]	17 (44.7)	10 (45.5)	0.999
Pre-clamping Serum Lactate (mmol/L)	1.0±0.4	1.5±1.7	0.139
Post-unclamping Serum Lactate (mmol/L)	1.8±0.7	1.8±0.7	0.849
Serum Lactate difference (mmol/L) [e]	0.8±0.5	0.7±0.7	0.478
Post- unclamping intraoperative diuresis (ml/kg)	1.0±0.6	1.3±1.0	0.117
Intra-operative blood loss (ml/kg)	12.0±7.0	10.5±5.1	0.380
Patients with post-operative complications; N (%)[a]	10 (26.3)	5 (22.7)	0.333
Pre-operative serum Creatinine (mg/dL)	1.0±0.3	1.0±0.3	0.973
48h post-operative serum Creatinine (mg/dL)	1.1±0.4	1.0±0.4	0.301
Δ serum Creatinine [f]	0.2±0.4	0.0±0.2	0.067
ICU admission; N (%)[a]	3 (7.9)	1 (4.5)	0.666
Hospital length-of-stay (days) [g]	4.3±4.4	3.8±1.6	0.615

Measures are reported as mean ± SD or number, N (percentage, %), as appropriate.
Paired T Test performed.
[a] Mann Whitney test performed.
[b] Δ MAP, computed as: ΔMAP= (MAP at Ao Clamp – MAP at Ao Unclamp)/ MAP at Ao Clamp
[c] Δ CI, computed as: ΔCI= (CI at Ao Clamp – CI at Ao Unclamp)/ CI at Ao Clamp
[d] Δ CPI, computed as: ΔCPI= (CPI at Ao Clamp – CPI at Ao Unclamp)/ CPI at Ao Clamp
[e] Serum lactate difference ($sLac_{diff}$), computed as: $sLac_{diff}$ = (sLac Unclamp – sLac Clamp)
[f] Δ serum Creatinine (Δ sCr), computed as: Δ sCr= (sCr 48h post-op – sCr pre-op) / sCr pre-op
[g] We excluded one case who stayed in Hospital >30 days due to a not aortic-surgery related complication (i.e. abdominal wall haematoma)
Abbreviations: Ao-XC, Aortic cross-clamping; Ao-UC, Aortic unclamping; Ao, Aortic (Clamp or Unclamp).
