# Supplementary figures and images for: The Cardiac Power Index during Abdominal Open Aortic Surgery: Intraoperative Insights into the Cardiac Performance—A Retrospective Observational Analysis
Source: J Pers Med. 2022 Oct 12;12(10):1705. doi: 10.3390/jpm12101705 (PMC9605046; doi:10.3390/jpm12101705)

**Supplemental Figure S1 – Study flow chart**

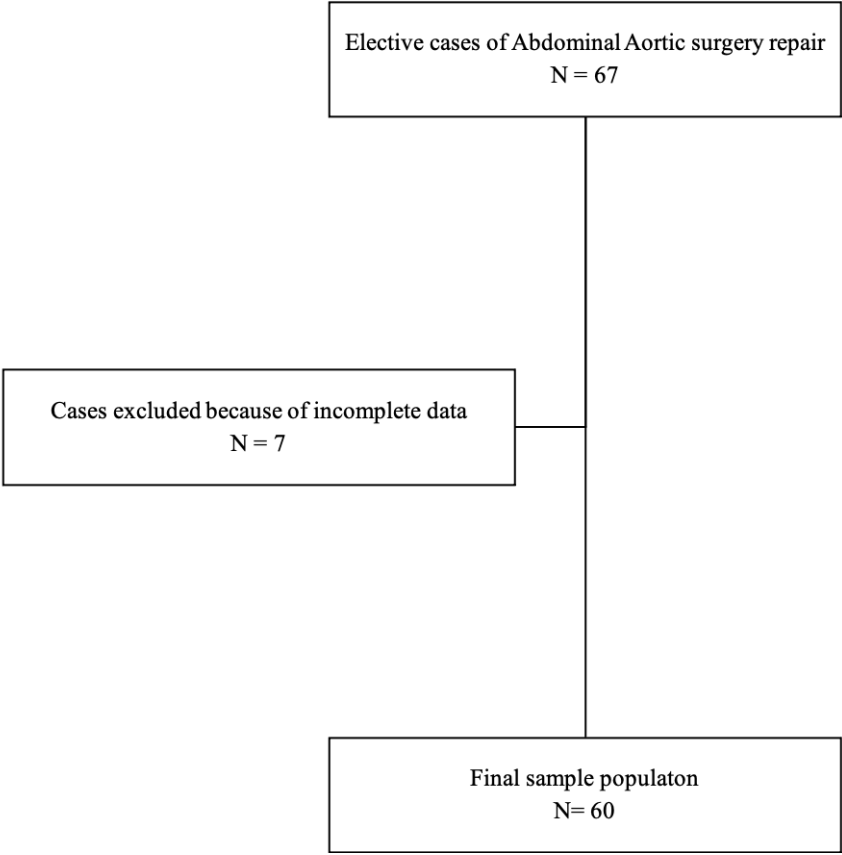

Supplement: Supplementary file 1 [file jpm-12-01705-s001.zip › Supplemental Figure S1.pdf]

**Supplemental Figure S2. CPI and age**

*Abbreviations.*

CPI, Cardiac Power Index

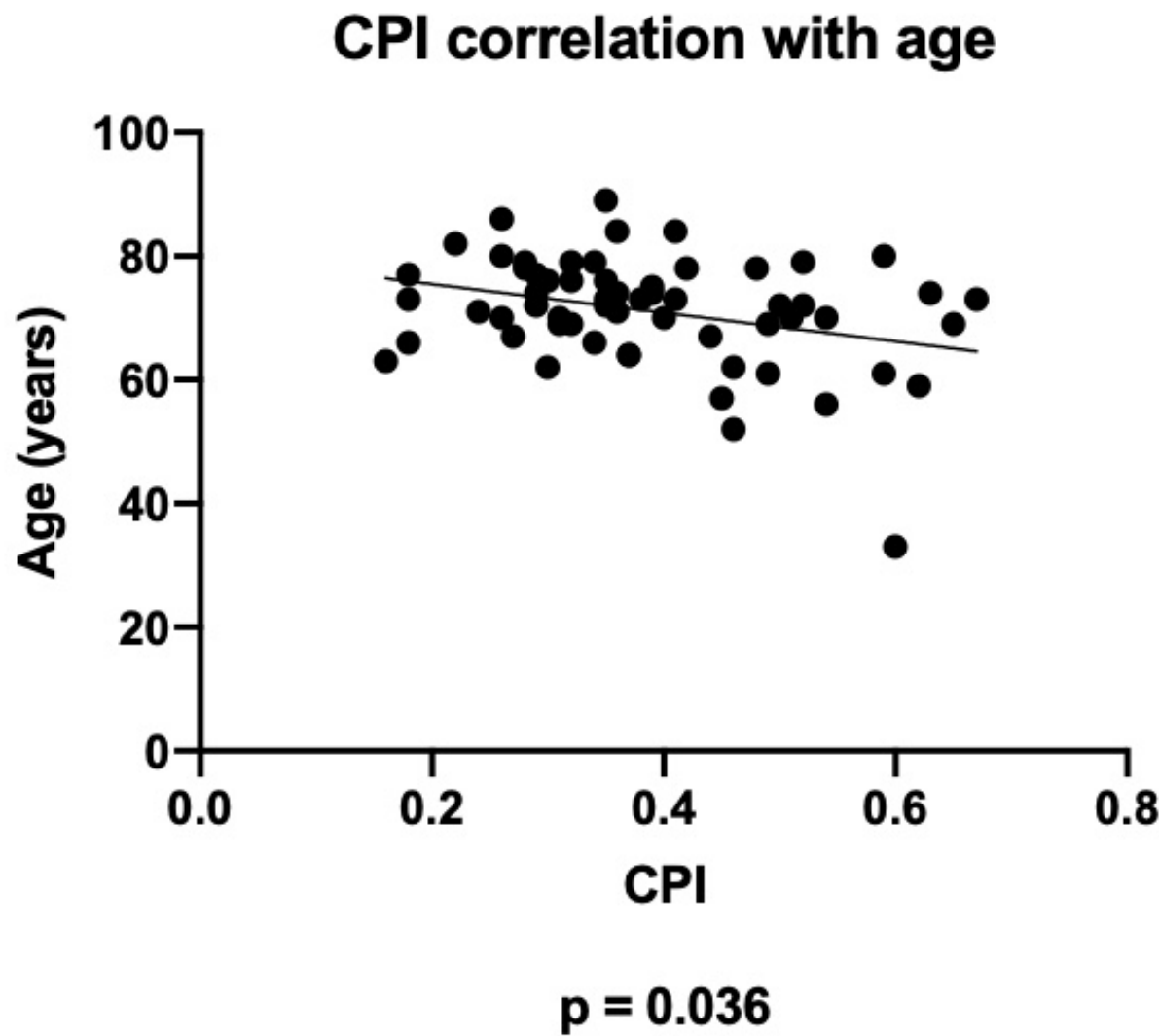

Supplement: Supplementary file 1 [file jpm-12-01705-s001.zip › Supplemental Figure S2.pdf]
